# Pathway Identification on the Contribution of Home Garden Vegetables to Improve Nutritional Status of Children

**DOI:** 10.1002/fsn3.70371

**Published:** 2025-06-05

**Authors:** Hailemariam Tekie Mahari, Zenebe Abraha Kahsay, Girmay Gebresamuel Abraha, Amanuel Zenebe Abraha

**Affiliations:** ^1^ Department of Food Science and Post‐Harvest Technology Mekelle University Mekelle Tigray Ethiopia; ^2^ Department of Agricultural and Resource Economics Mekelle University Mekelle Tigray Ethiopia; ^3^ Department of Land Resource Management and Environmental Protection Mekelle University Mekelle Tigray Ethiopia; ^4^ Institute of Climate and Society Mekelle University Mekelle Tigray Ethiopia

**Keywords:** children, income, pathways, principal component analysis, vegetables

## Abstract

Home‐garden vegetables could improve the nutritional status of children, but there are controversies on their impact and pathways through which they contribute to the improved nutritional status of children. The study was, therefore, conducted to analyze the pathways through which home‐garden vegetables contribute to the nutritional status of children 6–23 months in Southern Tigray, Ethiopia. A quasi‐experimental design was used to analyze the effect of the intervention using 382 sample households selected according to FANTA Sampling Guidelines. Face‐to‐face interviews were conducted using a structured questionnaire at baseline and endline surveys. Length‐for‐Age *Z* scores were computed using WHO Anthro‐2006 software. Descriptive statistics and Principal–Component Analysis were analyzed using STATA software version 12. Vegetable producers had a larger area cultivated and were significantly growing diverse crops than non‐producers. Households with greater than or equal to 1.5 ha of land were found to have the lowest but insignificant prevalence of child stunting. Though vegetable producers had a higher total annual income than non‐producers, there was no significant difference in the status of child stunting due to the annual income. The first principal component is highly associated with agriculture and farm income, while the next important variables associated with the components are household dietary diversity and income sources other than farm income. The production of vegetables and crop and income diversification from home gardens were found to be important pathways to improve food and nutrition security. Hence, concerned bodies need to promote sustainable production of diverse vegetables and income diversification in rural households to ensure food and nutrition security.

## Graphical abstract

The study was conducted to analyze the pathways through which home‐garden vegetables contribute to the nutritional status of children 6–23 months in Southern Tigray, Ethiopia, using quasi‐experimental design. A total of 382 sample households were used to analyze the effect of the intervention. The first principal component is highly associated with agriculture and farm income, while the next important variables associated with the components are household dietary diversity and income sources other than farm income. The production of vegetables and crop and income diversification from home gardens were found to be important pathways to improve food and nutrition security. Concerned bodies need to promote sustainable production of diverse vegetables and income diversification in the rural households to ensure food and nutrition security.

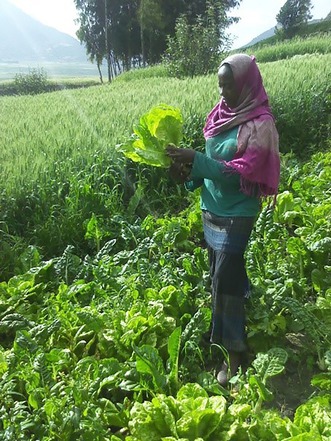

## 1 Introduction

Growth in the agricultural sector has contributed strongly to economic growth in Ethiopia over the past decades—yet increasing staple crop yields and investing in agricultural production has not resolved enough the high rates of child stunting (USAID 2021; FDRE 2018), which remains a major public health problem in Ethiopia (EPHI 2023). As a result, a multisectoral approach that comprises three pathways—nutrition‐specific, nutrition‐sensitive, and infrastructure interventions was—employed in Ethiopia through the Food and Nutrition Policy for the coordinated implementation of nutritional interventions to tackle nutrition and nutrition‐related health problems (FMoH 2016a; Ahmed et al. 2023).

Multi‐sectoral approaches and coordination mechanisms such as nutrition‐sensitive interventions alongside the nutrition‐specific interventions are believed to play a pivotal role in addressing the devastating impacts of malnutrition (FAO 2017; Wordofa and Sassi 2020). More particularly, nutrition‐sensitive agriculture is employed to maximize the positive impacts of the food system on nutrition outcomes (Agitew et al. 2023; Fanzo et al. 2014) through the pathways of agricultural food production, agricultural income, women's empowerment (Graaf et al. 2018; WFP 2017), and also nutrition‐related knowledge and strengthening local institutions (Agitew et al. 2024). However, the pathways from agriculture to nutrition are evolving and dynamic and are not always linear (Kadiyala et al. 2014). Hence, there is a need to describe potential pathways and relationships between factors to improve our understanding of how agriculture can best contribute to improved nutrition (Hanieh et al. 2019; Mwangome et al. 2024).

As part of the nutrition‐sensitive agriculture, home gardening could influence diet and nutrition outcomes through multiple pathways, including increased household availability of nutrient‐rich foods through own production and increased income and access to food in markets through sale of produce (SPRING 2014; Ruel et al. 2018). Impact evaluations of home garden interventions have significantly increased household vegetable production and consumption in Bangladesh (Baliki et al. 2019), and significantly increased the frequency of children's vegetable consumption and reduced anemia among mothers and children in Nepal (Schreinemachers et al. 2020).

Dorward (2013) explained that the relations between agriculture and nutrition are eminently complex; the risks vary depending on the nature and context of the intervention, with economic growth and development. Similarly, Pandey et al. (2016) indicated that agricultural growth is not a sufficient condition for addressing the problem of malnutrition because its impact is location specific and depends on the state of the economy and other socio‐demographic conditions. Striessnig and Bora (2020) and Husseini et al. (2018) also described that stunting is highly correlated with poorer socio‐economic status and environmental conditions. Hence, as nutrition interventions alone are insufficient, the importance of ongoing efforts to foster nutrition‐sensitive programs and approaches that address the underlying determinants of malnutrition is well justified (UNICEF 2019). Furthermore, Casanovas et al. (2013) encouraged the development of multi‐sectoral plans to deal with stunting on a national scale by combining direct nutrition interventions with strategies concerning health, family planning, water supply and sanitation, and other factors that affect the risk of stunting.

It is clear that different countries, regions, and localities have different types of cultures, feeding behaviors, nutritional knowledge, and other socio‐economic issues. This implies that the same agricultural intervention, such as home gardening, can have different nutritional impacts in different regions, depending on cultural practices, baseline malnutrition levels, market access, and caregiver knowledge. Thus, the importance of and contribution to nutritional status through home garden vegetables, especially during the first 2 years of human life, need to be studied based on the context of the study areas to understand the pathways that matter most and track intermediate indicators, which are often more sensitive to change. Therefore, the present study was conducted with the objective of analyzing the pathways through which home garden vegetables contribute to the nutritional status of children 6–23 months of age.

## 2 Materials and Methods

### 2.1 Description of the Study Areas

**FIGURE 1:**
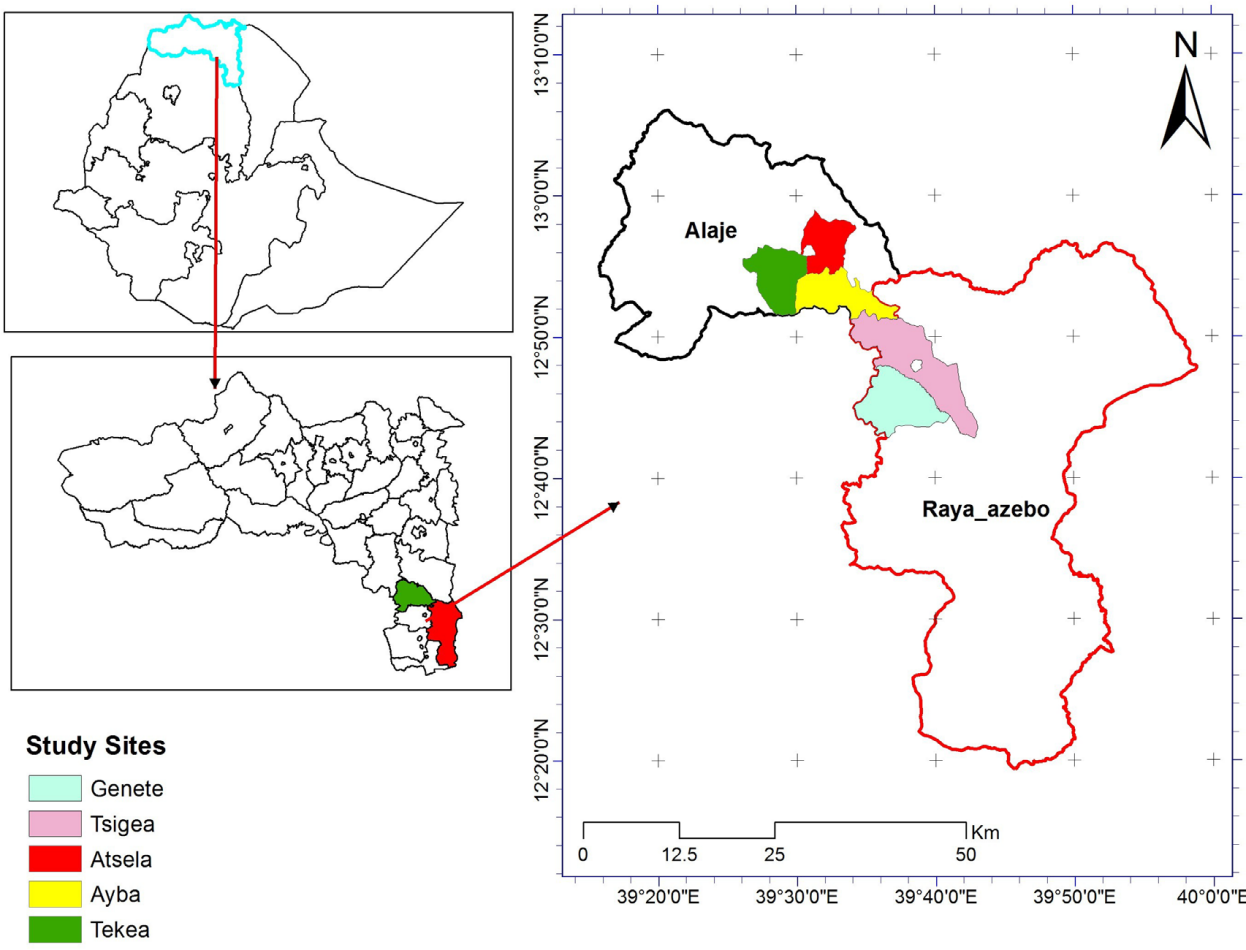
Map of the study areas.

The study was conducted in the southern zone of Tigray, where it is suitable for the production of different types of vegetables owing to its favorable climate and better access to surface and ground water sources. But, unfortunately, the status of vegetable production and consumption in the study areas is generally low. Five representative *Tabias* (villages) namely Genete and Tsigea from the Raya‐Azebo district, and Ayba, Atsela, and Tek'a from Emba‐Alaje district were selected as study sites considering the households' better experience in home‐garden vegetable production. The study areas were purposively selected with the help of the local experts. The study areas are geographically indicated in Figure 1.

### 2.2 Study Design and Sampling

The study was conducted using quasi‐experimental design to determine the pathways through which home garden vegetables contribute to the nutritional status of children aged 6–23 months. A total of 382 households were included in the study using the double proportion population formula outlined by FANTA Sampling Guidelines (Magnani 1999).

### 2.3 Data Collection

Data on socio‐economic variables, food security status of the households, and anthropometric data were collected during the study period (2019–2020). Data were collected by face‐to‐face interviews at baseline and end line survey using a structured questionnaire.

Socio‐economic variables collected through a structured household survey and included in the analysis were sex of the household head (categorized as male [= 1] and female [= 0]), total area cultivated (total land area under cultivation in hectares at the time of data collection), number of vegetables produced (the total number of types of vegetables produced during the last growing season), crop diversity (The number of different crops grown by the household, categorized according to their classification such as cereals, legumes, tubers, and other food categories), tropical livestock unit\TLU (livestock holdings based on the Tropical Livestock Unit system, which standardizes the size of different types of livestock to a common scale), and household income (the household income categorized as annual farm income, annual off‐farm income, annual on‐farm income, and total annual income).

The variables collected to reflect the food security status of households were based on Household Dietary Diversity Score (HDDS) and Household Food Insecurity Access Scale (HFIAS). HDDS was determined using the standard 24‐h recall technique. The tool inquires about 16 food groups, which are aggregated to 12 groups for analysis, as supported by FAO (2010). The respondents were asked to recall all foods and drinks consumed by the household members in the previous 24 h. Foods consumed outside the home but not prepared at home were not included. HFIAS is a standard indicator of food security measurements with a nine‐question tool developed by Food and Nutrition Technical Assistance (FANTA) to assess household food insecurity occurring within the previous month (Nsabuwera et al. 2015).

Anthropometric data were also collected to determine the nutritional status of children. Baseline and end line surveys of anthropometric measurements were collected to determine the nutritional status using length‐for‐age of children 6–21 months of age. The length (height) of a child was measured using a horizontal wooden length board in a recumbent position and read to the nearest 0.1 cm. The equipment needed to measure the length was placed on a flat surface, and measurement was conducted by trained health extension workers.

### 2.4 Data Processing and Analysis

Descriptive statistics were used to determine the frequencies and percentages of the socio‐economic characteristics of the study participants and status of child stunting using STATA software version 12.

Stunting measured by comparing the length and age of a child was expressed as standard deviation units from the median for the reference group and was compared to the 2006 WHO growth reference. Chi2 test was used to determine the percentage distribution of child stunting of the vegetable producer and non‐producer households based on the socioeconomic variables. Principal Component Analysis (PCA), a statistical technique that creates a small number of components from a set of correlated variables, was also used to establish the relationship between selected variables. Nutritional factors are very complex, and PCA reduces this complexity, allowing researchers to focus on a few key components that explain most of the variance due to the effect of explanatory variables. A *p*‐value < 0.05 was considered for statistical significance. A *p*‐value < 0.05 was considered for statistical significance.

## 3 Results

### 3.1 Socio‐Economic Characteristics and Child Stunting

Most of the vegetable producer and non‐producer households had cultivated land in the range of 0.5–1.0 ha (Table 1). Vegetable producers had a larger area cultivated compared to vegetable non‐producer households. Most of the vegetable producers grow a single type of vegetable in home gardens and significantly grow diverse crops compared to non‐producers, where most of them produced two types of crops, including vegetables. About 15% of vegetable producer and 29% of non‐producer households did not have any kind of livestock at the end line survey.

**TABLE 1:** Socio‐economic characteristics of the vegetable producer and non‐producer households.

Variables	Description	Base line (%)		End line (%)	
		VP	VNP	VP	VNP
Total area cultivated (ha)	No land	3.19	24.62	9.38	18.04
	< 0.5	25.53	21.92	17.71	20.00
	(0.5–1.0)	37.23	36.15	33.33	36.86
	(1.0–1.5)	20.21	13.08	26.04	17.25
	≥ 1.5	13.83	4.23	13.54	7.84
Number of vegetables produced	< 2	73.41	na	92.71	na
	≥ 2	26.6	na	7.3	na
Crop diversity	0	4.26	26.26	9.38	18.04
	1	13.83	30.5	21.88	32.94
	2	41.49	34.36	46.88	36.08
	3	35.11	6.95	17.71	9.41
	4	5.32	1.93	4.17	3.53
Tropical livestock unit (TLU)	0	17.02	29.23	14.58	28.63
	< 1	11.70	11.92	16.67	11.76
	(1–2)	15.96	16.15	15.63	11.37
	(2–3)	19.15	17.69	12.50	20.00
	≥ 3	36.17	25.00	40.63	28.24
Annual farm income (Birr)	0.00	9.57	35.77	10.42	27.45
	< 10,742	31.91	32.31	33.33	38.43
	10742–14742	18.09	13.08	7.29	10.59
	> 14742	40.43	18.85	48.96	23.53
Annual off‐farm income (Birr)	0.00	63.83	60.77	75.00	70.20
	< 0725	2.13	3.08	0.00	0.39
	0725–4725	17.02	16.54	2.08	7.84
	> 4725	17.02	19.62	22.92	21.57
Annual non‐farm income (Birr)	0.00	69.15	60.08	56.25	54.51
	< 11961	9.57	10.47	12.50	16.86
	11961–15961	4.26	5.43	4.17	7.06
	> 15961	17.02	24.03	27.08	21.57
Total annual income (Birr)	0.00	1.06	2.69	0.00	1.96
	< 23428	51.06	63.46	43.75	61.96
	23428–35428	9.57	8.85	20.83	13.73
	> 35428	38.30	25.00	35.42	22.35
Sex of head of household	MHH	96.81	88.85	91.67	83.92
	FHH	3.19	11.15	8.33	16.08
LAZ of children	Normal	81.40	78.75	56.98	52.89
	Stunted	18.60	21.25	43.02	47.11

Abbreviations: na, not applicable; VNP, vegetable non‐producers; VP, vegetable producers.

Income was determined and categorized based on the median income of households. Though most of the vegetable producer households generate an income greater than 14742 Birr (441 USD) in a year from the farm they cultivated, there were still many who generate an income less than 10742 Birr (321 USD) for the same period. However, most of the vegetable non‐producers generate an annual income of less than 10742 Birr from the farm they cultivated, while there were many who do not generate an income from their farms. In fact, about 25% of the vegetable non‐producers do not have cultivated land at the baseline survey. For both vegetable producers and non‐producers, there were no off‐farm and non‐farm income generated by most of the respondents, which shows that farm income is the main source of income. For the majority of the respondents in both groups, the total annual income generated was less than 23428 Birr in a year. But higher annual income was generated by the vegetable producers compared to the vegetable non‐producers (Table 1).

There was no significant association between vegetable producer and non‐producer households on the prevalence of child stunting, though child stunting seems to reduce with increasing size of cultivated land, number of vegetables produced, and crop diversity (Table 2). However, the prevalence of child stunting increases with increasing TLU, though results were inconsistent and insignificant. In both of the survey times, vegetable non‐producer households with greater than or equal to 1.5 ha of land were found to have the lowest but insignificant prevalence of child stunting.

**TABLE 2:** Percentage distribution of stunted children based on selected socio‐economic variables.

Variables	Description	Base line			End line		
		VP	VNP	chi^2^	VP	VNP	chi^2^
Total area cultivated (ha)	No land	12.50	31.37	0.266^a^ 0.158^b^ 0.064^c^	13.51	12.28	0.773^a^ 0.762^b^ 0.617^c^
	< 0.5	31.25	19.61		21.62	28.95	
	(0.5–1.0)	25.00	37.25		18.92	36.84	
	(1.0–1.5)	18.75	11.76		32.43	17.54	
	≥ 1.5	12.50	0.00		13.51	4.39	
Number of vegetables produced	< 2	93.75	na	0.087^a^ na^b^ na^c^	94.60	na	0.774^a^ na^b^ na^c^
	≥ 2	6.25	na		5.41	na	
Crop diversity	0	12.50	31.37	0.057^a^ 0.280^b^ 0.056^c^	13.51	12.28	0.242^a^ 0.000^b^ 0.000^c^
	1	25.00	33.33		18.92	24.56	
	2	31.25	29.41		35.14	44.74	
	3	25.00	1.96		27.03	14.04	
	4	6.25	3.92		5.41	4.39	
Tropical livestock unit (TLU)	0	6.25	27.45	0.388^a^ 0.619^b^ 0.915^c^	13.51	21.93	0.874^a^ 0.214^b^ 0.280^c^
	< 1	6.25	17.65		16.22	13.16	
	(1–2)	25.00	13.73		13.51	14.04	
	(2–3)	25.00	21.57		16.22	23.68	
	≥ 3	37.50	19.61		40.54	27.19	
Annual farm income (Birr)	0.00	12.50	47.06	0.285^a^ 0.054^b^ 0.024^c^	13.51	22.81	0.847^a^ 0.480^b^ 0.736^c^
	< 10742	37.50	31.37		29.73	42.11	
	10742–14742	18.75	7.84		10.81	12.28	
	> 14742	31.25	13.73		45.95	22.81	
Annual off‐farm income (Birr)	0.00	43.75	45.10	0.069^a^ 0.036^b^ 0.006^c^	70.27	74.56	0.282^a^ 0.065^b^ 0.310^c^
	< 0725	0.00	7.84		na	0.88	
	0725–4725	31.25	25.49		0.00	8.77	
	> 4725	25.00	21.57		29.73	15.79	
Annual non‐farm income (Birr)	0.00	68.75	49.02	0.359^a^ 0.049^b^ 0.027^c^	51.35	50.00	0.588^a^ 0.620^b^ 0.491^c^
	< 11961	0.00	9.80		13.51	21.93	
	11961–15961	0.00	5.88		5.41	7.02	
	> 15961	31.25	35.29		29.73	21.05	
Total annual income (Birr)	0.00	0.00	0.00	0.410^a^ 0.284^b^ 0.212^c^	na	0.88	0.572^a^ 0.935^b^ 0.905^c^
	< 23428	43.75	58.82		43.24	64.91	
	23428–35428	6.25	13.73		18.92	11.40	
	> 35428	50.00	27.45		37.84	22.81	
Sex of head of household	MHH	100.00	86.27	0.402^a^ 0.455^b^ 0.617^c^	91.89	85.09	0.993^a^ 0.988^b^ 0.947^c^
	FHH	0.00	13.73		8.11	14.91	

Abbreviations: VNP, vegetable non‐producer; VP, vegetable producer.

^a^ Within VP.

^b^ Within VNP.

^c^ Between VP and VNP.

Though the prevalence of child stunting was not significantly associated with annual farm income for vegetable producers and non‐producers, increasing farm income of the households explains the reduction in stunting of children at the age of 6–23 months in the current study, especially for vegetable non‐producers (Table 2). However, there was no consistent and significant difference between vegetable producer and non‐producer households based on the other sources of annual income. Furthermore, there were unpredicted results that the lowest prevalence of child stunting was observed at the lowest total annual income generated, reflecting that a significant proportion of the income earned was not used for household food consumption.

### 3.2 Principal Component Analysis (PCA)

The first four principal components together explain about 65.95% and 61.97% of the variance for VP and VNP, respectively (Table 3). The first principal component, which accounted for about 27% of the total variance in vegetable producers, had a strong positive loading for the number of vegetables produced, total area cultivated, crop diversity, tropical livestock unit, farm income, and the sex of HH head while it was negatively correlated with off‐farm income and HFIAS. For vegetable non‐producers, the first principal component accounted for about 24% of the total variance. This component had a strong positive loading for total area cultivated, crop diversity, tropical livestock unit, and farm income and negative loadings with non‐farm income. It was correlated most strongly with the total area cultivated and crop diversity.

**TABLE 3:** PCA of the socio‐economic and nutritional characteristics of households.

Variable	PC 1		PC 2		PC 3		PC 4	
	VP	VNP	VP	VNP	VP	VNP	VP	VNP
Sex of household head	0.371	0.257	0.194	0.193	−0.136	0.481	−0.437	0.155
No. of vegetables produced	0.486	na	−0.090	na	0.616	na	0.251	na
Total area cultivated	0.778	0.715	−0.208	−0.034	−0.250	0.199	0.016	0.167
Crop diversity	0.825	0.836	−0.146	0.012	0.194	−0.184	0.176	−0.118
TLU	0.713	0.618	−0.213	−0.066	−0.279	0.361	−0.151	0.300
Farm income	0.598	0.647	0.093	0.316	0.376	0.221	0.267	−0.099
Off‐farm income	−0.527	−0.279	−0.247	−0.282	0.269	0.550	−0.194	−0.314
Non‐farm income	−0.108	−0.339	0.931	0.877	0.085	0.057	−0.099	−0.122
Total annual income	0.166	−0.109	0.867	0.927	0.359	0.229	0.036	−0.206
HDDS	0.264	−0.185	0.445	0.411	−0.636	−0.086	0.140	0.582
HFIAS	−0.508	−0.233	−0.142	−0.267	0.034	0.523	0.538	−0.363
LAZ	−0.082	−0.196	0.169	−0.084	−0.426	0.471	0.628	0.443
Eigenvalue	3.179	2.841	2.091	2.131	1.514	1.361	1.131	1.104
% variance	26.49	23.67	17.42	17.76	12.61	11.34	9.43	9.20

Abbreviations: na, not applicable; VNP, vegetable non‐producers; VP, vegetable producers.

The second principal component was found to explain about 17% and 18% of the total variance in vegetable producer and non‐producer households, respectively. Non‐farm income and total annual income were strongly associated with the component in both groups. In addition, HDDS was moderately correlated with the component in both groups and farm income weakly associated with vegetable non‐producers.

In the third component, a positive association was observed on the number of vegetables produced, farm income, total annual income, and a negative correlation with HDDS and LAZ for vegetable producers, while for the vegetable non‐producers, the component was positively correlated with the sex of the HH head, TLU, off‐farm income, HFIAS, and LAZ, with a total variance of about 13% and 11% for the vegetable producers and non‐producers, respectively.

Though the total variance to explain the association of the principal component with the variables is small in the fourth component, there was a positive association with HDDS and LAZ, but these variables were negatively associated with the sex of the HH head in vegetable producers. This component was dominated by positive loadings of TLU, HDDS, and LAZ in vegetable non‐producers. These variables were, however, negatively correlated with off‐farm income and HFIAS in the last component for vegetable non‐producers.

In the first two components, LAZ is not associated with the variables listed in the table, but to some extent, there was a positive correlation with HDDS and HFIAS and a negative correlation with the number of vegetables produced, farm income, total annual income, and sex of the HH head in vegetable producer households. For vegetable non‐producers, LAZ was positively associated with the sex of the HH head, TLU, off‐farm income, HFIAS, and HDDS, while it was negatively associated with off‐farm income and HFIAS. There was no significant association between the sex of the head of the household and the intermediate dietary factors such as HDDS and HFIAS in the vegetable non‐producer households. However, there was a significant relationship among the variables, where higher HDDS and lower HFIAS were observed in MHHs compared to FHHs in the vegetable producer households. Generally, the table shows that the first component that explains most of the variance is highly associated with agriculture and food security‐related issues, including income from the farm, while the next important variables associated with the components are HDDS and income sources other than farm income.

## 4 Discussion

### 4.1 Socio‐Economic Characteristics and Child Stunting

Though it was insignificant, there was a trend of reduction in child stunting with increased land size and number of vegetables produced, which could be due to the possible reduction of poverty, which positively influences the welfare of rural households as a result of increased diversity and availability of foods from their own farm. This could be taken as one pathway that links home garden production with nutrition. It was also confirmed by Eyasu (2020) that the size of cultivated land positively increases the welfare of households in Ethiopia. Hence, vegetable production from one's own home garden has the prospect to contribute towards the reduction of child stunting.

Increasing crop diversity also reduces the chance of occurrence of child stunting for both vegetable producer and non‐producer households. Moreover, there was a significant difference between the two groups on the prevalence of child stunting. However, vegetable production did not show any significant difference in the length‐for‐age of children at both survey times. This indicates that it is the crop diversity that matters most for length‐to‐age of children than access to vegetable production alone. According to Dangura and Gebremedhin (2017) and Beyero et al. (2015), there was a significant positive association between ownership of home gardens and children's dietary diversity. Promoting crop diversity including vegetables within the existing plot of land can benefit households and children in particular, either in the form of direct consumption or increased income from sale of vegetables. Hence, crop diversity is another possible pathway of home garden vegetable production to improve nutritional status of children. According to CSA (2018), grain crops dominate Ethiopia's agricultural production. Among grain crops, cereals account for up to 88% of production, followed by pulses (10%) and oil seeds (2%). But vegetables and fruits account only for a small share of Ethiopia's crop production (2.2% and 2.1%, respectively). Though growth in the production of grains, and particularly cereals, is key for rural livelihoods in Ethiopia, crop diversity within the available land is more important to improve household dietary intake, which was also confirmed by Gbenga et al. (2020), Singh et al. (2020), and Djokoto et al. (2017). As a result, crop diversification, as another identified pathway, should be encouraged, as it was also reflected by Charlie et al. (2020) as a promising strategy to improve dietary diversity and nutritional status.

Tropical livestock unit (TLU) was higher for vegetable producers than non‐producers, but the prevalence of child stunting was not significantly lower in vegetable producers. The failure to reduce child stunting with increased TLU in this study could be due to limited use of their animal products for household food consumption of better‐quality diets. Unlike the results of the current study, livestock ownership was found to be positively correlated with LAZ of children (Beyene et al. 2019; Headey et al. 2017). On the other hand, Headey and Hirvonen (2016) showed that keeping poultry in the same house with people is found to negatively affect LAZ of children. So, another reason for the negative effect of TLU on the length‐for‐age of children could be the presence of livestock excreta and the poor hygienic conditions of the rural community that affect health, which in turn affects the nutritional status of children. As indicated by Hoddinott et al. (2015), owning a single cow has the potential to increase LAZ (between 0.25 and 0.47 standard deviations) and reduce stunting levels (between 6% and 13%). Hence, it is essential to further investigate the gaps that limit the proper utilization of livestock for improved nutritional status of children.

Though agriculture has been the backbone of the Ethiopian economy, the mean annual farm income of the farmers in the study area was Birr 14689.39 (USD 439.41) and Birr 9169.59 (USD 274.29) for vegetable producers and non‐producers, respectively, while an average small family farm in Ethiopia generates a gross annual income of about USD 1246 (FAO 2018). This shows that there are limited agricultural products produced for sale in the study areas, probably due to low agricultural productivity, which could be associated with the poor implementation of market‐oriented agricultural strategies that have been introduced by the agriculture extension system. There was a significant difference in the farm income of vegetable producers and non‐producers, but this could not bring a significant difference in the prevalence of child stunting. Unlike the results of the current study, Gebremedhin et al. (2017) indicated that households with home gardens are twice as likely to provide diversified food to improve the nutritional status of children. Hence, vegetable production, if properly implemented, not only makes vegetable products available for own consumption but also serves as a good source of income for food and other resources. The insignificant and inconsistent association of the farm income and prevalence of child stunting in this study could therefore be due to a lack of childcare as children get older, regardless of the significant difference observed in the farm income of vegetable production. A similar finding was reported by Benfica and Kilic (2016) on the association of income diversification and high‐quality diets in rural Malawi. Let alone income diversification, though statistically insignificant, Productive Safety Net Program (PSNP) participant households receiving cash were found to have a lower prevalence of stunting due to higher levels of consumption of oils and fats, legumes, and dairy products in the Tigray region (Debela et al. 2015). Hence, the problem of reduction of child stunting at higher farm income in this study indicates that farm income could not guarantee nutritional security as far as other influencing factors are being improved.

Child stunting was found to be significantly different between vegetable producers and non‐producers at the baseline survey, and the prevalence of child stunting was highest in households with no farm income for both groups. Similar to this study, Anang and Yeboah (2019) and Babatunde and Qaim (2009) reported that the prevalence of child stunting is lower in households with off‐farm income than in households without, as it might help to contribute higher food production and farm income by easing capital constraints. However, Huang et al. (2009) signified a negative impact of off‐farm income, as working off the farm could potentially reduce household food availability due to the competition for family labor between farm and off‐farm work. He also pointed out that off‐farm work could especially be at the expense of livestock and horticultural on‐farm activities, as these are particularly labor‐intensive. Hence, in case off‐farm income is not used for food consumption in areas where cereal is the dominant food group, it might limit the availability of diverse foods such as vegetables, which are good sources of micronutrients. At the endline survey, lower child stunting was observed in households that have higher off‐farm income, though it was insignificant at different off‐farm income levels. According to Anang and Yeboah (2019), income from off‐farm activities is an alternative source of income for the agricultural sector, which may be used to finance agricultural production. So, the lower prevalence of stunting at higher off‐farm income could be due to higher agricultural output per unit of land as a result of income diversification. Off‐farm and non‐farm activities, which were observed to be very low in the study areas, are therefore essentially important, as Danso‐Abbeam (2020), Dagunga et al. (2018), and Chirwa et al. (2017) have also widely recognized: income diversification strategies have a positive impact on the welfare of households. Similarly, Astatike and Gazuma (2019) indicated that diversification into off‐farm and/or non‐farm activities has become a surviving strategy for most rural farm households because farming as a principal source of income has failed to assure sufficient livelihood for most rural farming households in developing countries, especially in Sub‐Sahara Africa. Hence, income diversification has the potential to increase farm investment, leading to higher productivity and then positive impacts on nutrition.

Vegetable producers had significantly higher total annual income than non‐producers at the 10% level of significance, which indicates that home garden vegetable production is an important pathway to nutrition through income generation. But both non‐farm and total annual income did not show any significant effect on the status of child stunting, though there was a significant difference (*p* = 0.027) between vegetable producers and non‐producers on the prevalence of child stunting based on non‐farm income at the baseline survey. The mean total annual income generated by the vegetable producers and non‐producers of the respondent farmers in this study was Birr 27499.34 (USD 822.6) and Birr 22515.91 (USD 673.5), respectively, which is much lower than Ethiopia's per capita income of USD 862 reported by UNDP (2018). As the purchase of a high‐quality diet could be a challenge, the lower annual income might be the reason for the lack of positive effect on length‐for‐age of children in this study. According to the recent Situation Analysis of the Nutrition Sector (SITAN) study in Ethiopia, there was an association between poverty and stunting, where children from the lowest wealth quintile were found to be stunted (FMoH 2016b).

Bachewe et al. (2016) revealed that more than 70% of the income of rural households in Ethiopia comes from crop production, while non‐farm income accounts for only 6% of rural households. This shows that Ethiopia's rural non‐farm economy is still at an early stage in farming systems and its development. Though there are demographic and socioeconomic reasons to limit non‐farm income sectors, the most important constraints for Ethiopian households are access to finance, access to markets, and transportation (CSA 2017). Hence, the lower annual income in the study areas reflects that there is low understanding or poor policy concern on non‐farm sectors in the rural areas. As a result, there was no consistent association of total income generation and prevalence of child stunting. This could also be due to farmers' inability to invest in nutritious and diverse foods during the off‐season. Therefore, there is a need to promote sustainable production of diverse foods and income diversification. Moreover, the use of income to invest in food, health, and care resources, especially for children, is essential when there are seasonal shocks. USAID (2019) shows that children under 2 years of age are one of the groups most nutritionally vulnerable and that they need special provision of diet, health services, and care for their growth and development. To overcome the problem, concerned bodies need to look forward to the barriers and challenges aforementioned and design better strategies to help farmers become productive per unit of land in terms of crop and income diversity, which will in turn assure food and nutrition security.

### 4.2 Principal Component Analysis

The first principal component that most explains the relationship between the variables indicates that there is a strong correlation among the variables related to agricultural production and farm income in the first component, but these variables were not associated with the nutrition indicators such as HDDS and LAZ. This reflects that the agricultural activities implemented in the study areas were not aligned to improve dietary diversity and the nutritional status of children. The poor agricultural productivities in terms of quality and quantity of products could be contributing factors to the limited impact of the variables on nutrition. HFIAS was negatively associated with the agriculture‐related activities in vegetable producers but not in non‐producers, which shows that agricultural activities were aligned to improve food security in vegetable producer households.

There are research findings that revealed dietary diversity slightly higher for FHHs (Olabisi et al. 2021; Jateno et al. 2023). However, there are also many findings that showed MHHs with higher dietary diversity (Argaw et al. 2021; Kabeta et al. 2023; Aliyo et al. 2022). This explains that nutritional outcomes such as dietary diversity are context specific. According to Murendo et al. (2018) and Olabisi et al. (2021), farm diversification and production of more food categories have a positive and significant association with household dietary diversity, where MHHs are more advantaged. Whereas, FHHs face many social and cultural barriers that limit their participation in development activities (Negesse et al. 2020; Sheahan and Barrett 2017; Hiruy et al. 2023) and are less productive compared to male managed plots (Covarrubias 2015). This implies that the better intermediate food security indicators such as HDDS and HFIAS in MHHs in the current study may stem from differences in better resources of land and other agricultural inputs to improve crop and income diversity compared to FHHs.

The most important variables that are highly associated in the second component are related to HDDS and income sources other than farm income for both vegetable producers and non‐producers. But HFIAS and LAZ were not associated with this component. This indicates that it is own production that matters most for the farmers than income sources other than farm income to improve food security and LAZ in the study areas, which could be as a result of lack of use of income for consumption of high‐quality foods. According to Al Jawaldeh et al. (2020), stunting prevalence was found to be inversely associated with country income, which is unlike the findings reported in this study.

## 5 Conclusion and Recommendation

Agriculture and economic related variables such as land size, crop diversity, vegetable production and number of vegetables produced, TLU, and income were not well incorporated to reduce child stunting though the tendency to improve food and nutritional status of households observed in the study was an indication that these variables would have the prospect to positively improve the nutritional status of children. Vegetable production could not have a direct effect on child stunting but there are indirect opportunities to improve the food security status of households.

Crop and vegetable diversification is a promising strategy to improve dietary diversity and nutritional status if complemented with income diversification and women's empowerment, especially economic and knowledge on child feeding practices. Hence, governmental and non‐governmental organizations need to give particular consideration to the production of vegetables, crop, and income diversification from own home gardens in the rural community as possible pathways to improve their livelihoods and then improve food and nutrition security. Moreover, they need to encourage the promotion and introduction of post‐harvest technologies in the rural households to make sure that there is year‐round availability of nutritious and diverse foods, implying the importance of vegetable production for own consumption and income generation.

## Ethics Statement

Ethical clearance was obtained from the Research Ethics Review Committee (RERC) of the Institute of Climate and Society, Mekelle University. Moreover, the study was approved by the local offices at district and sub‐district levels. After the approval from local officials, each participant consented orally to participate in the study and was informed of the objectives of the study, their rights to refuse participation, stop participating at any time of the interview, and to skip specific questions or topics they do not want to answer.

## Conflicts of Interest

The authors declare no conflicts of interest.

## Data Availability

The data that support the findings of this study are available on request from the corresponding author.

## References

[fsn370371-bib-0001] Agitew, G. , Z. Berhanie , and S. Gebremedhin . 2023. Nutrition Sensitivity of Smallholder Agricultural Production in Northwest Ethiopia.

[fsn370371-bib-0002] Agitew, G. , Z. Berhanie , and S. Gebremedhin . 2024. “Production Pathway of Smallholders to Nutrition‐Sensitive Agriculture in Northwest Ethiopia.” Discover Agriculture 2: 25.

[fsn370371-bib-0003] Ahmed, K. , F. Oqbo , T. Tegegne , H. Dalton , A. Arora , and A. Ross . 2023. “Interventions to Improve the Nutritional Status of Children Under 5 Years in Ethiopia: A Systematic Review.” Public Health Nutrition 26: 3147–3161. 37905557 10.1017/S1368980023002410 PMC10755407

[fsn370371-bib-0004] Al Jawaldeh, A. , R. Doggui , E. Borghi , et al. 2020. “Tackling Childhood Stunting in the Eastern Mediterranean Region in the Context of COVID‐19.” Children (Basel) 7, no. 11: 239. 33227997 10.3390/children7110239 PMC7699289

[fsn370371-bib-0005] Aliyo, A. , W. Golicha , and A. Fikrie . 2022. “Household Dietary Diversity and Associated Factors Among Rural Residents of Gomole District, Borena Zone, Oromia Regional State, Ethiopia.” Health Services Research and Managerial Epidemiology 9: 23333928221108033. 35720258 10.1177/23333928221108033 PMC9201295

[fsn370371-bib-0006] Anang, B. T. , and R. W. N. Yeboah . 2019. “Determinants of off‐Farm Income Among Smallholder Rice Farmers in Northern Ghana: Application of a Double‐Hurdle Model.” Advances in Agriculture 2019: 7246176.

[fsn370371-bib-0007] Argaw, T. L. , E. Phimister , and D. Roberts . 2021. “From Farm to Kitchen: How Gender Affects Production Diversity and the Dietary Intake of Farm Households in Ethiopia.” Journal of Agricultural Economics 72, no. 1: 268–292.

[fsn370371-bib-0008] Astatike, A. A. , and E. G. Gazuma . 2019. “The Impact of Off‐Farm Activities on Rural Household Income in Wolaita Zone, Southern Ethiopia.” Journal of World Economic Research 8, no. 1: 8–16.

[fsn370371-bib-0009] Babatunde, R. O. , and M. Qaim . 2009. Impact of Off‐Farm Income on Food Security and Nutrition in Nigeria, Proceedings of the German Development Economics Conference, Frankfurt a.M. 2009, No. 27, Verein für Socialpolitik, Ausschuss Für Entwicklungsländer, Göttingen.

[fsn370371-bib-0010] Bachewe, F. , G. Berhane , B. Minten , and A. S. Taffesse . 2016. Non‐Farm Income and Labor Markets in Rural Ethiopia, No. ESSP Working Paper 90, IFPRI and Ethiopian Development Research Institute (EDRI), Washington, D.C. and Addis Ababa, Ethiopia.

[fsn370371-bib-0011] Baliki, G. , T. Brück , P. Schreinemachers , and M. N. Uddin . 2019. “Long‐Term Behavioural Impact of an Integrated Home Garden Intervention: Evidence From Bangladesh.” Food Security 11, no. 6: 1217–1230.

[fsn370371-bib-0012] Benfica, R. , and T. Kilic . 2016. The Effects of Smallholder Agricultural Involvement on Household Food Consumption and Dietary Diversity: Evidence from Malawi. IFAD (International Fund for Agricultural Development).

[fsn370371-bib-0013] Beyene, S. , M. S. Willis , M. Mamo , B. Legesse , T. Regassa , and T. Tadesse . 2019. “Nutritional Status of Children Aged 0–60 Months in Two Drought‐Prone Areas of Ethiopia.” South African Journal of Clinical Nutrition 33, no. 4: 152–157.

[fsn370371-bib-0014] Beyero, M. , H. Judith , and L. Amanda . 2015. Leveraging Agriculture for Nutrition in East Africa (LANEA) Country Report–Ethiopia. FAO/IFPRI.

[fsn370371-bib-0015] Casanovas, M. C. , C. Lutter , N. Mangasaryan , et al. 2013. “Multisectoral Interventions for Healthy Growth.” Maternal & Child Nutrition 9, no. 2: 46–57. 24074317 10.1111/mcn.12082 PMC6860720

[fsn370371-bib-0016] Charlie, C. N. , F. E. Benjamin , and T. N. Meredith . 2020. “Global Relationships Between Crop Diversity and Nutritional Stability.” bioRxiv 12, no. 1: 5310. 10.1038/s41467-021-25615-2 PMC8423801 34493729

[fsn370371-bib-0017] Chirwa, C. W. , D. Makoka , B. B. Maonga , and D. H. Ng'ong'ola . 2017. “Impact of Malawi's Farm Income Diversification Programme on Household Welfare: Empirical Evidence From Eleven Districts.” In MaSSP Working Paper 20. IFPRI.

[fsn370371-bib-0018] Covarrubias, K. A. 2015. The Role of Crop Diversity in Household Production and Food Security in Uganda: A Gender‐differentiated Analysis. Wageningen UR: LEI.

[fsn370371-bib-0019] CSA . 2017. Integrated Surveys on Agriculture Ethiopia Socioeconomic Survey (ESS), Central Statistical Agency and Living Standards Measurement Study (LSMS), World Bank.

[fsn370371-bib-0020] CSA . 2018. Agricultural Sample Survey (2018), Central Statistical Agency, Addis Ababa.

[fsn370371-bib-0021] Dagunga, G. , D. S. Ehiakpor , I. K. Parry , and G. Danso‐Abbeam . 2018. “Determinants of Income Diversification Among Maize Farm Households in the Garu‐Tempane District.” Review of Agricultural and Applied Economics 21, no. 1: 55–63.

[fsn370371-bib-0022] Dangura, D. , and S. Gebremedhin . 2017. “Dietary Diversity and Associated Factors Among Children 6‐23 Months of Age in Gorche District, Southern Ethiopia: Cross‐Sectional Study.” BMC Pediatrics 17, no. 1: 1–7. 28068965 10.1186/s12887-016-0764-x PMC5223415

[fsn370371-bib-0066] Danso‐Abbeam, G. , G. Dagunga , and D. S. Ehiakpor . 2020. “Rural Non‐Farm Income Diversification: Implications on Smallholder Farmers’ Welfare and Agricultural Technology Adoption in Ghana.” Heliyon 6, no. 11: e05393. 33210002 10.1016/j.heliyon.2020.e05393 PMC7658696

[fsn370371-bib-0023] Debela, B. L. , S. Gerald , and T. H. Stein . 2015. “Does Ethiopia's Productive Safety Net Program Improve Child Nutrition?” Food Security 7, no. 6: 1273–1289.

[fsn370371-bib-0024] Djokoto, J. G. , V. Afari‐Sefa , and A. Addo‐Quaye . 2017. “Vegetable Diversification in Cocoa‐Based Farming Systems Ghana.” Agriculture & Food Security 6: 6. 10.1186/s40066-016-0082-4.

[fsn370371-bib-0025] Dorward, A. 2013. “How Can Agricultural Interventions Contribute in Improving Nutrition Health and Achieving the MDGs in Least Developed Countries? Working Paper, CeDEP (Centre for Development, Environment and Policy).” In SOAS, University of London and Leverhulme Centre for Integrative Research in Agriculture and Health, 22. SOAS and LCIRAH. 10.1159/000354946 24504210

[fsn370371-bib-0026] EPHI . 2023. National Food and Nutrition Strategy Baseline Survey: Key Findings Preliminary Report. Ethiopian Public Health Institute.

[fsn370371-bib-0027] Eyasu, A. M. 2020. “Determinants of Poverty in Rural Households: Evidence From North‐Western Ethiopia.” Cogent Food & Agriculture 6, no. 1: 1823652.

[fsn370371-bib-0028] Fanzo, J. , M. Cohen , T. Sparling , T. Olds , and M. Cassidy . 2014. The Nutrition Sensitivity of Agriculture and Food Policies: A Synthesis of Eight Country Case Studies. United Nations System, Standing Committee on Nutrition.

[fsn370371-bib-0064] FAO (Food and Agriculture Organization of the United Nations) . 2010. Guidelines for Measuring Household and Individual Dietary Diversity. FAO.

[fsn370371-bib-0029] FAO . 2017. Nutrition‐Sensitive Agriculture and Food Systems in Practice: Options for Intervention. Food and Agriculture Organization of the United Nations.

[fsn370371-bib-0030] FAO . 2018. Small Family Farms Country Factsheet, Ethiopia.

[fsn370371-bib-0031] FDRE . 2018. Food and Nutrition Policy. Federal Democratic Republic of Ethiopia.

[fsn370371-bib-0032] FMoH . 2016a. The Seqota Declaration Committed to Ending Stunting in Children Under Two by 2030. Addis Ababa: Ethiopia Federal Ministry of Health.

[fsn370371-bib-0033] FMoH . 2016b. Situation Analysis of the Nutrition Sector in Ethiopia 2000–2015. Ethiopian Federal Ministry of Health, UNICEF, and European Commission Delegation, Addis Ababa, Ethiopia.

[fsn370371-bib-0034] Gbenga, O. , H. I. Opaluwa , S. O. Adedeji , and S. Abdulrahman . 2020. “Effect of Crop Diversity on Rural Farming Households' Dietary Diversity.” Journal of Asian Rural Studies 4, no. 2: 218–229.

[fsn370371-bib-0035] Gebremedhin, S. , B. Kaleab , B. Tilahun , et al. 2017. “Predictors of Dietary Diversity in Children Ages 6 to 23 Mo in Largely Food‐Insecure Area of South Wollo, Ethiopia.” Nutrition 33, no. January: 163–168. 27499206 10.1016/j.nut.2016.06.002

[fsn370371-bib-0036] Graaf, M. , G. Newton , N. Verhart , et al. 2018. Metrics and Programing in Nutrition‐Sensitive Agriculture. Netherlands Working Group on International Nutrition.

[fsn370371-bib-0037] Hanieh, S. , S. Braat , J. A. Simpson , et al. 2019. “The Stunting Tool for Early Prevention: Development and External Validation of a Novel Tool to Predict Risk of Stunting in Children at 3 Years of Age.” BMJ Global Health 4, no. 6: e001801. 10.1136/bmjgh-2019-001801 PMC6861113 31798990

[fsn370371-bib-0038] Headey, D. , and K. Hirvonen . 2016. “Is Exposure to Poultry Harmful to Child Nutrition? An Observational Analysis for Rural Ethiopia.” PLoS One 11, no. 8: e0160590. 27529178 10.1371/journal.pone.0160590 PMC4986937

[fsn370371-bib-0039] Headey, D. , N. Phuong , K. Sunny , R. Rahul , R. Marie , and M. Purnima . 2017. “Is Exposure to Animal Feces Harmful to Child Nutrition and Health Outcomes? A Multicountry Observational Analysis.” American Journal of Tropical Medicine and Hygiene 96, no. 4: 961–969. 27994099 10.4269/ajtmh.16-0270 PMC5392649

[fsn370371-bib-0040] Hiruy, H. N. , J. Barden‐O'Fallon , F. Mitiku , and E. Millar . 2023. “Association of Gender‐Related Factors and Household Food Security in Southwest Oromia, Ethiopia: Evidence From a Cross‐Sectional Study.” Agriculture & Food Security 12, no. 1: 26.

[fsn370371-bib-0041] Hoddinott, J. , H. Derek , and D. Mekdim . 2015. “Cows, Missing Milk Markets, and Nutrition in Rural Ethiopia.” Journal of Development Studies 51, no. 8: 958–975.

[fsn370371-bib-0042] Huang, J. , Y. Wu , and S. Rozelle . 2009. “Moving off the Farm and Intensifying Agricultural Production in Shandong: A Case Study of Rural Labor Market Linkages in China.” Agricultural Economics 40, no. 2: 203–218.

[fsn370371-bib-0043] Husseini, M. , M. K. Darboe , S. E. Moore , H. M. Nabwera , and A. M. Prentice . 2018. “Thresholds of Socio‐Economic and Environmental Conditions Necessary to Escape From Childhood Malnutrition: A Natural Experiment in Rural Gambia.” BMC Medicine 16: 199. 30382849 10.1186/s12916-018-1179-3 PMC6211595

[fsn370371-bib-0044] Jateno, W. , B. A. Alemu , and M. Shete . 2023. “Household Dietary Diversity Across Regions in Ethiopia: Evidence From Ethiopian Socio‐Economic Survey Data.” PLoS One 18, no. 4: e0283496. 37018284 10.1371/journal.pone.0283496 PMC10075445

[fsn370371-bib-0045] Kabeta, T. , R. Holst , B. Wondafrash , A. Frigessi , and M. K. Gebremariam . 2023. “Determinants of Household Dietary Diversity in Rural Ethiopia: A Household Panel Study.” Journal of Agriculture and Food Research 12: 100550.

[fsn370371-bib-0046] Kadiyala, S. , J. Harris , D. Headey , S. Yosef , and S. Gillespie . 2014. “Agriculture and Nutrition in India: Mapping Evidence to Pathways.” Annals of the New York Academy of Sciences 1331: 43–56. 25098622 10.1111/nyas.12477

[fsn370371-bib-0047] Magnani, R. 1999. Sampling Guide. Food and Nutrition Technical Assistance Project, Academy for Educational Development.

[fsn370371-bib-0048] Murendo, C. , B. Nhau , K. Mazvimavi , T. Khanye , and S. Gwara . 2018. “Nutrition Education, Farm Production Diversity, and Commercialization on Household and Individual Dietary Diversity in Zimbabwe.” Food & Nutrition Research 62: 10–29219. 10.29219/fnr.v62.1276 PMC5965157 29849533

[fsn370371-bib-0049] Mwangome, M. , M. Ngari , D. Brals , et al. 2024. “Stunting in the First Year of Life: Pathway Analysis of a Birth Cohort.” PLOS Global Public Health 4, no. 2: e0002908. 38363746 10.1371/journal.pgph.0002908 PMC10871522

[fsn370371-bib-0050] Negesse, A. , D. Jara , H. Temegen , et al. 2020. “The Impact of Being of the Female Gender for Household Head on the Prevalence of Food Insecurity in Ethiopia: A Systematic Review and Meta‐Analysis.” Public Health Reviews 41: 15. 32518705 10.1186/s40985-020-00131-8 PMC7275294

[fsn370371-bib-0065] Nsabuwera, V. , B. Hedt‐Gauthier , M. Khogali , et al. 2015. “Making Progress Towards Food Security: Evidence from an Intervention in Rural Districts of Rwanda.” Public Health Nutrition 19, no. 7: 1296–1304. 26246309 10.1017/S1368980015002207 PMC4825097

[fsn370371-bib-0051] Olabisi, M. , H. O. Obekpa , and L. S. O. Liverpool‐Tasie . 2021. “Is Growing Your Own Food Necessary for Dietary Diversity? Evidence From Nigeria.” Food Policy 104: 102144.

[fsn370371-bib-0052] Pandey, V. L. , S. D. Mahendra , and U. Jayachandran . 2016. “Impact of Agricultural Interventions on the Nutritional Status in South Asia: A Review.” Food Policy 62: 28–40. 27478297 10.1016/j.foodpol.2016.05.002 PMC4952527

[fsn370371-bib-0053] Ruel, M. T. , A. R. Quisumbing , and M. Balagamwala . 2018. “Nutrition‐Sensitive Agriculture: What Have We Learned So Far?” Global Food Security 17: 128–153.

[fsn370371-bib-0054] Schreinemachers, P. , G. Baliki , R. M. Shrestha , et al. 2020. “Nudging Children Toward Healthier Food Choices: An Experiment Combining School and Home Gardens.” Global Food Security 26: 100454. 33324538 10.1016/j.gfs.2020.100454 PMC7726313

[fsn370371-bib-0055] Sheahan, M. , and C. B. Barrett . 2017. “Ten Striking Facts About Agricultural Input Use in Sub‐Saharan Africa.” Food Policy 67: 12–25. 28413243 10.1016/j.foodpol.2016.09.010 PMC5384438

[fsn370371-bib-0056] Singh, S. , A. D. Jones , R. S. DeFries , and M. Jain . 2020. “The Association Between Crop and Income Diversity and Farmer Intra‐Household Dietary Diversity in India.” Food Security 12: 369–390. 10.1007/s12571-020-01012-3.

[fsn370371-bib-0057] SPRING . 2014. Linking Agriculture and Nutrition: A Guide to Context Assessment Tools. USAID Strengthening Partnerships, Results, and Innovations in Nutrition Globally (SPRING) Project.

[fsn370371-bib-0058] Striessnig, E. , and J. K. Bora . 2020. “Under‐Five Child Growth and Nutrition Status: Spatial Clustering of Indian Districts.” Spatial Demography 8: 63–84.

[fsn370371-bib-0059] UNICEF . 2019. The State of the World's Children 2019. In: Children, Food and Nutrition: Growing Well in a Changing World. UNICEF. https://www.unicef.org/reports/state‐of‐worlds‐children‐2019.

[fsn370371-bib-0060] USAID . 2019. Nutrition‐Sensitive Agriculture: Applying the Income Pathway: Technical Guidance Brief.

[fsn370371-bib-0061] USAID . 2021. Agriculture and Food Security. Feed the Future Value Chain Activity, Ethiopia. USIAD. https://www.usaid.gov/ethiopia/agriculture‐and‐food‐security/feed‐future.

[fsn370371-bib-0062] WFP . 2017. “Guidance for Nutrition‐Sensitive Programing.” World Food Program 2017: 1–54.

[fsn370371-bib-0063] Wordofa, M. G. , and M. Sassi . 2020. “Impact of Agricultural Interventions on Food and Nutrition Security in Ethiopia: Uncovering Pathways Linking Agriculture to Improved Nutrition.” Cogent Food & Agriculture 6, no. 1: 1724386.

